# Synaptic proteomics decode novel molecular landscape in the brain

**DOI:** 10.3389/fnmol.2024.1361956

**Published:** 2024-04-25

**Authors:** Yuki Ito, Sayaka Nagamoto, Tetsuya Takano

**Affiliations:** ^1^Division of Molecular Systems for Brain Function, Institute for Advanced Study, Medical Institute of Bioregulation, Kyushu University, Fukuoka, Japan; ^2^Division of Integrated Omics, Medical Research Center for High Depth Omics, Medical Institute of Bioregulation, Kyushu University, Fukuoka, Japan; ^3^Department of Neurophysiology, Keio University School of Medicine, Tokyo, Japan; ^4^PRESTO, Japan Science and Technology Agency, Saitama, Japan

**Keywords:** synapse, BioID, APEX, proteomics, astrocyte, neuron, TurboID, Split-TurboID

## Abstract

Synapses play a pivotal role in forming neural circuits, with critical implications for brain functions such as learning, memory, and emotions. Several advances in synaptic research have demonstrated the diversity of synaptic structure and function, which can form thousands of connections depending on the neuronal cell types. Moreover, synapses not only interconnect neurons but also establish connections with glial cells such as astrocytes, which play a key role in the architecture and function of neuronal circuits in the brain. Emerging evidence suggests that dysfunction of synaptic proteins contributes to a variety of neurological and psychiatric disorders. Therefore, it is crucial to determine the molecular networks within synapses in various neuronal cell types to gain a deeper understanding of how the nervous system regulates brain function. Recent advances in synaptic proteome approaches, such as fluorescence-activated synaptosome sorting (FASS) and proximity labeling, have allowed for a detailed and spatial analysis of many cell-type-specific synaptic molecules *in vivo*. In this brief review, we highlight these novel spatial proteomic approaches and discuss the regulation of synaptic formation and function in the brain. This knowledge of molecular networks provides new insight into the understanding of many neurological and psychiatric disorders.

## Introduction

A human brain consists of approximately 86 billion neurons, interconnected through an intricate network of around 100 trillion synapses. In addition to neurons, glial cells such as astrocytes are involved in the formation of synapses, particularly evident in structures termed “tripartite synapses.” These cellular networks are crucial for the establishment of neural circuits underlying highly complex brain functions such as memory, learning, and emotion. Despite their incredibly small volume within neural cells, synapses play a key role in determining neuronal functionality. These synapses exhibit diverse functions as well as shapes, depending on the composition of thousands of proteins that vary across different neuronal types. Importantly, recent advancements in gene profiling have demonstrated that the abnormality of the synaptic protein is highly associated with psychiatric and neurological disorders, thus defining it as a “synaptopathies” (Lepeta et al., 2016; Luo et al., 2018; Yoshino et al., 2021). Thus, it is crucial to identify spatial molecular networks within synapses across specific neuronal cell types to understand how the nervous system governs the variety of brain functions. However, it remains a significant challenge to comprehend how a wide variety of synaptic molecules control each neuronal connectivity and functioning in the brain.

Over the past several decades, numerous researchers have attempted to isolate synapses to uncover the intricate functions orchestrated by synaptic molecules. Synaptic molecules have been isolated and analyzed using a variety of biochemical approaches such as cell fractionation, density gradient centrifugation (e.g., synaptosomes), immunoprecipitation, and affinity chromatography followed by liquid chromatography–tandem mass spectrometry (LC–MS/MS). These proteomic analyses have shed light on the components of synaptic proteins, as demonstrated by a comprehensive study that identified 2,876 proteins across 41 *in vivo* interactomes from mouse cerebral cortex, revealing the extensive landscape of the core-scaffold machinery within the postsynaptic density (Li et al., 2017). In particular, the identification of several key components of synaptic molecules, such as the NMDA receptor complex and PSD95 complex, has emerged as crucial discoveries in current synaptic research (Husi et al., 2000; Dosemeci et al., 2007; Fernández et al., 2009; Delint-Ramirez et al., 2010; Xu et al., 2021). Other major synaptic protein complexes were later elucidated, including SHANKs (Lee et al., 2017), SynGAP (Krapivinsky et al., 2004), PSD-93 (Zhang et al., 2020), FMRP (Schenck et al., 2001; Pasciuto and Bagni, 2014; Zhang et al., 2019), and metabotropic glutamate receptors (Farr et al., 2004; Francesconi et al., 2009; Ramos et al., 2012; Pandya et al., 2016). Additionally, further research has revealed that protein networks of small GTPase proteins control synapse formation (Wilkinson et al., 2017). Despite the effectiveness of these approaches, deciphering the molecular composition of synapses at the cellular level *in vivo* has been technically challenging due to the inability to maintain the spatial resolution indicating their originating cell types. In recent years, innovative approaches such as fluorescence-activated synaptosome sorting (FASS) and proximity labeling (BioID and APEX) approaches have been developed to facilitate the analysis of the spatial proteome, unveiling the cell-type-specific molecular composition within specific subcellular sites such as synapse and synaptic cleft *in vivo* (Biesemann et al., 2014; Han et al., 2018; Takano and Soderling, 2021). Additionally, we have recently developed novel *in vivo* cell-surface BioID approaches, namely TurboID-surface and Split-TurboID. These techniques have proven highly effective in elucidating the molecular network of cell-type-specific connections such as neurons and astrocytes (Takano et al., 2020). Here, this mini-review emphasizes the application of cutting-edge spatial proteomic approaches to investigate the molecular networks of cell-type-specific synapse compartments in the brain. Additionally, we discuss the current insights into synapse formation and function *in vivo*. These fascinating studies will contribute to our understanding of individual neural circuit structure and function as well as the pathomechanisms of psychiatric and neurological disorders.

## Spatial synaptic proteome by fluorescence activated synaptosome sorting

One of the most significant breakthroughs in the synaptic proteome has been the isolation of synaptosomes from the brain and the subsequent comprehensive proteomic analysis of their molecular components. In 1964, Dr. Whittaker and his colleagues first successfully purified the synaptic compartment termed “synaptosome” from brain tissue through density-gradient centrifugation (Whittaker et al., 1964). The purification of synaptosomes has enabled extensive studies on the molecular composition and structure of synapses. However, these conventional synaptosome studies faced difficulties in purifying cell-type-specific synaptic compartments *in vivo*.

Fluorescence activated synaptosome sorting (FASS) is a powerful approach that improves the purification of conventional synaptosomes (Biesemann et al., 2014). The synaptosomes are fluorescently labeled by a transgenic approach with fluorescent proteins or the application of dyes specifically targeting synaptic membranes. After fluorescent labeling, they are isolated by flow cytometry and flowed by LC–MS/MS (Biesemann et al., 2014; Apóstolo et al., 2020; Paget-Blanc et al., 2022). Thus, FASS technique allows for the analysis of cell-type-specific synaptic compartments (Biesemann et al., 2014). Using VGLUT1^VENUS^ knock-in mice, fluorescent glutamatergic synaptosomes (VGLUT1-positive excitatory synapse) were selectively purified and identified 163 synaptic proteins in the mouse forebrain (Biesemann et al., 2014). A notable finding of this study is the discovery of FXYD6 and Tpd52 as novel glutamatergic synapse components (Biesemann et al., 2014). Moreover, FASS approach was utilized to analyze the cell-surface protein composition of mossy fiber synapses in the hippocampus that govern microcircuit connectivity related to cognitive function (Apóstolo et al., 2020). Mossy fiber synaptosomes were purified by fluorescent labeling with Nectin-3 and FM4-64 membrane dye, followed by isolation using a fluorescent cell sorter (Apóstolo et al., 2020). Based on the approach, 77 proteins were identified as cell surface proteins of the mossy fiber synapse *in vivo* (Apóstolo et al., 2020). Among these proteins, this study reveals that IgSF8 is a crucial regulator of CA3 microcircuit connectivity and function (Apóstolo et al., 2020). FASS has recently also been employed for the synaptic molecular mapping of dopaminergic neurons *in vivo* (Paget-Blanc et al., 2022). Dopaminergic synapses were labeled by the expression of adeno-associated viral (AAV) vectors carrying Cre-dependent EGFP in the dopaminergic neurons (DAT-Cre transgenic mouse). EGFP-labeled dopaminergic synaptosomes were purified and then sorted using fluorescent cell sorters. It was found that 57 proteins specifically enriched at the dopaminergic synapse (Paget-Blanc et al., 2022). Interestingly, these dopaminergic synaptosomes contain multiple synaptic compartments including glutamatergic, GABAergic, or cholinergic synapses, that form “dopamine hub synapses” (Paget-Blanc et al., 2022). The dopamine hub synapses potentially modulate dopamine volume transmission, which is crucial for reward processing and motor control. Thus, FASS approach enables spatial synaptic proteome analysis through improved isolation specificity for particular cell types in comparison to conventional synaptosome purification.

## Proximity labeling

**Figure 1:**
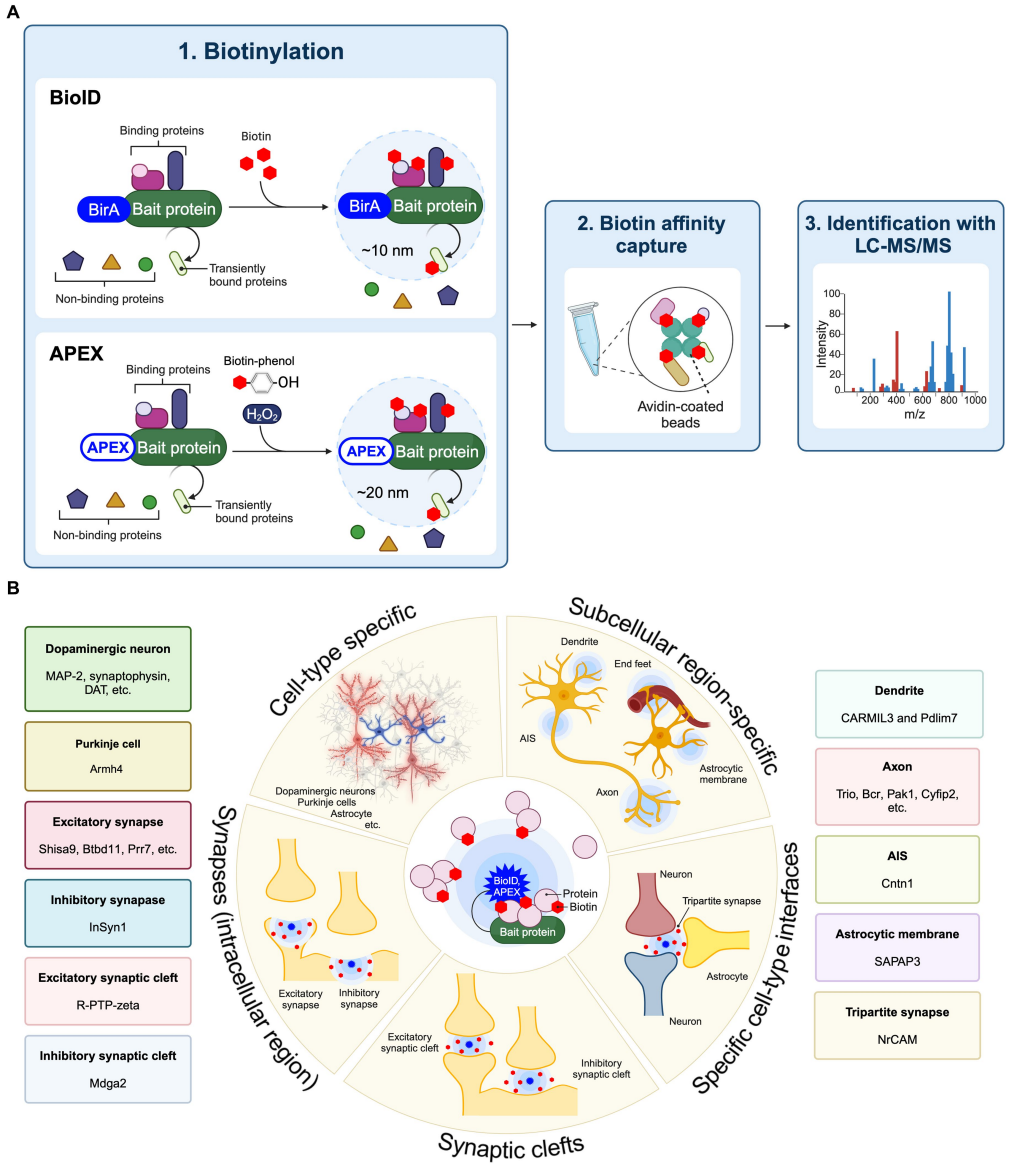
Application of proximity labeling to identify molecular landscape at specific cell type and subcellular site *in vivo*. **(A)** A schematic diagram and application of BioID and APEX. The protein of interest (bait) is fused with BirA*-R118G or APEX and expressed in cells. The enzyme biotinylates neighboring proteins of the bait protein. The biotinylated proteins are purified using avidin beads. Mass spectrometry is then utilized to identify proteins around the bait protein. **(B)** Proximity labeling approaches such as BioID and APEX enable the spatial proteomic analysis at cell-type-specific levels combined with subcellular localization, including individual types of synapses from neurons and astrocytes in the brain, using cell-type-specific AAVs or transgenic mouse lines. Proximity labeling can specifically target distinct cell types such as neurons and astrocytes to identify protein components through the cell-type-specific expression approaches *in vivo*. Additionally, proximity labeling can find protein networks within particular subcellular compartments, including the cell surface, dendritic spine, axon, axon initial segment (AIS), astrocytic membrane, end feet, and synapses by utilizing protein fusion targeted to these subcellular regions. At synapses, proximity labeling is capable of labeling intracellular synaptic proteins or synaptic cleft proteins by fusion with scaffold proteins or cell adhesion proteins. Split-TurboID also can decipher protein compositions between different cell types in the brain. Representative molecular landscapes that identified by these cell-type specific BioID or APEX are also shown.

Proximity labeling is a highly innovative approach to evaluating protein components present in specific cell types and subcellular localizations within cultured neurons as well as brain tissue (Han et al., 2018; Takano and Soderling, 2021). Proximity labeling is classified as either peroxidase-based proximity labeling or biotin ligase-based proximity labeling (BioID) (Han et al., 2018). Peroxidase-based proximity labeling utilizes ascorbate peroxidase derivatives like APEX or horseradish peroxidase (HRP) (Honke and Kotani, 2011; Rhee et al., 2013). By fusing these enzymes to a bait protein and introducing them into cells, they label tyrosine residues in proteins within a 10–20 nm range with biotin (Figure 1A). Following affinity purification with avidin-coated beads, the biotinylated proteins can be analyzed in more detail using LC–MS/MS to discover the spatial protein composition. Since the biotinylation labeling by HRP occurs in 1 min through biotin-phenol radical catalysis in the presence of hydrogen peroxide (H_2_O_2_), it is suitable for the analysis of spatial proteomes in living cells *in vitro* and *ex vivo*. Also, APEX catalyzes the oxidation and deposition of diaminobenzidine (DAB) and can also be used for electron microscopy imaging. BioID approach first utilized an *Escherichia coli*-derived mutant biotin ligase (BirA*-R118G), which produces reactive biotin (biotinoyl-5′-AMP) with an enhanced off-rate such that biotin covalently binds to exposed lysine residues of neighboring proteins within approximately 10 nm (Roux et al., 2012; Kim et al., 2016). There are several biotin ligases currently used in BioID approach, including BioID2, BASU, miniTurbo, TurboID, ultraID, microID, MicroID2, and AirID (Kim et al., 2016; Branon et al., 2018; Ramanathan et al., 2018; Kido et al., 2020; Johnson et al., 2022; Kubitz et al., 2022). Recently, BioID has been extensively applied to spatial proteome analysis in the brain and referred to as “*in vivo* BioID (iBioID)” (Uezu et al., 2016). iBioID possesses biocompatibility, high detection sensitivity, and spatial resolution capabilities, making it one of the most effective approaches for uncovering molecular landscapes specific to neuronal cell types and synapses. Therefore, we have summarized these approaches to elucidate the spatial proteome, which includes the molecular landscapes of synapses and the subcellular compartments of neurons and astrocytes, as well as the molecular mechanisms controlling synaptic formation and function in the brain (Figure 1B).

## Cell-type and synapse-type specific protein mapping

Proximity labeling allows for the isolation of protein components within excitatory and inhibitory synapses *in vivo*. Notably, the structural characteristics of inhibitory synapses make it challenging to access via conventional synaptosomal purification. Dr. Soderling and his colleagues first established *in vivo* BioID (iBioID) to investigate the proteomes of excitatory and inhibitory synapses in the brain using BirA*-R118G fused with postsynaptic scaffold proteins PSD-95 and gephyrin (Uezu et al., 2016) (Figure 1B; Table 1). In this study, AAV vectors carrying PSD95-BirA or gephyrin-BirA were expressed in mouse cortical and hippocampal neurons, and the biotin-labeled synaptic proteins were analyzed by LC–MS/MS. These proteomic approaches successfully identified 121 excitatory synaptic proteins and 181 inhibitory synaptic proteins, including both known and novel proteins. The important finding is that a novel inhibitory synaptic protein InSyn1 plays a critical role in inhibitory synapse formation and function through the interaction with the dystrophin complex *in vivo* (Uezu et al., 2016). This iBioID approach has facilitated the spatial proteome analysis within various neuronal intracellular compartments (Figure 1B). For example, the analysis of the dendritic spine proteome has been investigated using Rac-GAP (Wrp)-BirA and BioID2-synaptopodin (Spence et al., 2019; Falahati et al., 2022), while the axonal proteome has been explored using BioID2-Synapsin (O’Neil et al., 2021). Recently, the proteome of axon initial segment (AIS) has been detected using Neurofascin-HRP (Ogawa et al., 2023) (Figure 1B; Table 1). iBioID has also been applied to extensive interactome analyses of molecules associated with autism spectrum disorders (ASD) (Gao et al., 2023) (Table 1). In this study, a high-throughput genome-editing approach was used to target 14 high-confidence ASD genes (*Anks1b*, *Syngap1*, *Shank2*, *Shank3*, *Nckap1*, *Nbea*, *Ctnnb1*, *Lrrc4c*, *Iqsec2*, *Arhgef9*, *Ank3*, *Scn2a*, *Scn8a*, and *Hnrnpu*) for the insertion of a highly active mutant biotin ligase TurboID. These genes were selected for an iBioID-based proteome as spatially localized proteins at neuronal compartments such as the synapse, AIS, and nucleus. Comparative interactome analysis revealed a significant overlap of protein interactions among the targeted genes, with enrichment for ASD-related cellular functions. Additionally, this genome-editing mediated iBioID approach enables the investigation of native proteomes without relying on any overexpression methods *in vivo* (Gao et al., 2023). Therefore, these approaches will enable comprehensive analyses of synaptic-related molecules in physiological states and pathological conditions that lead to neurological disorders. iBioID approach has also been utilized for the cell-type-specific proteomic analysis of glutamatergic neurons and astrocytes in the brain using cell-type-specific AAV vectors or transgenic mouse lines (Sun et al., 2022) (Table 1). By using two different cell-type-specific promotors to express TurboID into the glutamatergic neurons (CaMKII promoter) and astrocytes (GFAP promoter) in the brain, more than 10,000 proteins were identified with excellent reproducibility (Sun et al., 2022). In addition to AAV-based expression approaches with TurboID or BioID2, neuron-specific (*Rosa26*^*TurboID*/*wt*^/CaMK2a) and astrocyte-specific (*Rosa26*^*TurboID*/*wt*^/Aldh1l1) TurboID knock-in mouse lines were established to investigate the cell-type-specific proteome *in vivo* (Rayaprolu et al., 2022) (Table 1). They identified over 2,000 proteins in each cell type, and more than 200 proteins that are different between neurons and astrocytes. Notably, MAPK signaling was elevated in neurons compared to astrocytes. These findings suggest the clarification of unique protein networks in CaMK2a-positive glutamatergic neurons and Aldh1l1-positive astrocytes, which are associated with their distinct cellular functions (Rayaprolu et al., 2022). Recently, BV2 microglial cells expressing cytoplasmic TurboID were analyzed to investigate their intracellular proteome (Sunna et al., 2023) (Table 1). Under lipopolysaccharide (LPS)-stimulated inflammatory conditions, more than 500 proteins showed differential abundance out of the 2,350 identified, demonstrating an increase in inflammatory proteins (Il1a, Irg1, Oasl1) and a decrease in anti-inflammatory proteins (Arg1, Mgl2) (Sunna et al., 2023). Moreover, the proteome specific to different cell types and subcellular locations was assessed using AAV vectors carrying either cytosolic-BioID2 or plasma membrane-BioID2 *in vivo* (Soto et al., 2023) (Figure 1B; Table 1). They were expressed in mouse striatal neurons (AAV-*hSynI*-BioID2 and AAV-*hSynI*-Lck-BioID2) and astrocytes (AAV-*GfaABC_1_D*-BioID2 and AAV-*GfaABC_1_D*-Lck-BioID2) (Soto et al., 2023). Using these approaches, they discovered a range of 500–1,800 proteins in the cytoplasm and plasma membrane of neurons and astrocytes. Furthermore, the subcellular proteomes of five distinct astrocyte subcellular compartments were explored *in vivo* (Soto et al., 2023) (Table 1). These include the end feet (AQP4-BioID2), astrocytic process (EZR-BioID2), extracellular glutamate uptake site (GLT1-BioID2), extracellular K^+^ homeostasis site (KIR4.1-BioID2) and astrocyte-astrocyte contacts (CX43-BioID2). These approaches were highly successful, revealing that SAPAP3 is specifically localized to the astrocytic process and involved in obsessive-compulsive disorder (OCD) and repetitive behaviors (Soto et al., 2023).

**Table 1:** Summary of proximity labeling approaches to decode molecular landscape in the brain.

Proximity labeling	Method (virus vectors or conditional knock-in mouse)	Brain region	Cell type	Subcellular compartment	The number of identified proteins	References
PSD95-BirA	AAV vector	Hippocampus	Neurons	Excitatory post-synapse	121	Uezu et al. (2016)
Gephyrin-BirA	AAV vector	Hippocampus	Neurons	Inhibitory post-synapse	181	Uezu et al. (2016)
Rac-GAP (Wrp)-BirA	AAV vector	Hippocampus	Neurons	Dendritic spine	60	Spence et al. (2019)
BioID2-synaptopodin	AAV vector	Hippocampus	Neurons	Dendritic spine	140	Falahati et al. (2022)
BioID2-Synapsin	AAV vector	Hippocampus	Neurons	Axon	200	O’Neil et al. (2021)
Neurofascin-HRP	AAV vector	Hippocampus	Neurons	Axon initial segment (AIS)	285	Ogawa et al. (2023)
ASD risk gene knock-in TurboID (HiUGE-iBioID)	Conditional knock-in mouse	Whole brain	Neurons	Synapse, AIS, and nucleus	1,252	Gao et al. (2023)
TurboID	AAV vector (CaMKII promoter)	Cortex	Neurons	Cytoplasm	9,919	Sun et al. (2022)
TurboID	AAV vector (GFAP promoter)	Cortex	Astrocytes	Cytoplasm	–	Sun et al. (2022)
TurboID	*Rosa26*^*TurboID*/*wt*^/Camk2a mice	Cortex, hippocampus, striatum/thalamus, pons/medulla, and cerebellum	Neurons	Cytoplasm	2,550 in total (including Rosa26^TurboID/wt^/Aldh1l1)	Rayaprolu et al. (2022)
TurboID	*Rosa26*^*TurboID*/*wt*^/Aldh1l1 mice	Cortex, hippocampus, striatum/thalamus, pons/medulla, cerebellum, and spinal cord	Astrocytes	Cytoplasm	2,550 in total (including Rosa26^TurboID/wt^/Camk2a)	Rayaprolu et al. (2022)
TurboID-NES	Lentivirus vector	Cultured microglia	Microglia	Cytoplasm	2,350	Sunna et al. (2023)
BioID2	AAV vector (*GfaABC_1_D* promoter)	Striatum	Astrocytes	Cytoplasm	–	Soto et al. (2023)
Lck-BioID2	AAV vector (*GfaABC_1_D* promoter)	Striatum	Astrocytes	Plasma membrane	–	Soto et al. (2023)
BioID2	AAV vector (human *SynI* promoter)	Striatum	Neurons	Cytoplasm	–	Soto et al. (2023)
Lck-BioID2	AAV vector (human *SynI* promoter)	Striatum	Neurons	Plasma membrane	–	Soto et al. (2023)
AQP4-BioID2	AAV vector (*GfaABC_1_D* promoter)	Striatum	Astrocytes	End feet	–	Soto et al. (2023)
EZR-BioID2	AAV vector (*GfaABC_1_D* promoter)	Striatum	Astrocytes	Astrocytic process	–	Soto et al. (2023)
GLT1-BioID2	AAV vector (*GfaABC_1_D* promoter)	Striatum	Astrocytes	Extracellular glutamate uptake site	–	Soto et al. (2023)
KIR4.1-BioID2	AAV vector (*GfaABC_1_D* promoter)	Striatum	Astrocytes	Extracellular K^+^ homeostasis site	–	Soto et al. (2023)
CX43-BioID2	AAV vector (*GfaABC_1_D* promoter)	Striatum	Astrocytes	Astrocyte-astrocyte contacts	–	Soto et al. (2023)
H2B-APEX2	Drd1-Cre mice	Striatum slice	D1-type of medium spiny neurons	Nucleus	2,191 and 2,332	Dumrongprechachan et al. (2021)
H2B-APEX2	A2a-Cre mice	Striatum slice	D2-type of medium spiny neurons	Nucleus	2,332	Dumrongprechachan et al. (2021)
APEX2-NES	Drd1-Cre mice	Striatum slice	D1-type of medium spiny neurons	Cytoplasm	2,191 and 2,332	Dumrongprechachan et al. (2021)
APEX2-NES	A2a-Cre mice	Striatum slice	D2-type of medium spiny neurons	Cytoplasm	2,332	Dumrongprechachan et al. (2021)
Lck-APEX2	Drd1-Cre mice	Striatum slice	D1-type of medium spiny neurons	Plasma membrane	2,191	Dumrongprechachan et al. (2021)
APEX2-NES	AAV vector, DAT-IRES-Cre mice	Ventral midbrain (VM) slice, medial forebrain bundle (MFB) slice, striatum slice	Dopaminergic neurons	Somatodendrites and axon terminal	1,449 proteins for VM, 702 proteins for MFB, 1,840 proteins for striatal samples	Hobson et al. (2022)
APEX	*Rbp4*^Cre^ mice	Cortical pyramidal neuron	Neurons	Axon	5,582	Dumrongprechachan et al. (2022)
HPR-Lrrtm1, HRP-Lrrtm2	Lentivirus vector	Cultured cortical neuron	Neurons	Glutamatergic excitatory synaptic cleft	199	Loh et al. (2016)
HRP-Slitrk3, HRP-Nlgn2	Lentivirus vector	Cultured cortical neuron	Neurons	GABAergic inhibitory synaptic cleft	42	Loh et al. (2016)
SynCAM 1-HRP	AAV vector	Cultured cortical neuron	Neurons	Excitatory synaptic cleft	39	Cijsouw et al. (2018)
HRP-CD2	*Drosophila*	Olfactory projection neurons in dissected brain	Neurons	Plasma membrane	20	Li et al. (2020)
iPEEL	Pcp2-Cre mice	Cerebellar slice	Purkinje cells	Plasma membrane	1,051	Shuster et al. (2022)
TurboID-surface	AAV vector (*GfaABC_1_D* promoter)	Cortex	Astrocytes	Plasma membrane	178	Takano et al. (2020)
Split-TurboID	AAV vector (human Synapsin I (*SynI*) promoter for N TurboID, *GfaABC_1_D* promoter for C TurboID)	Cortex	Neurons (N TurboID), astrocytes (C TurboID)	Tripartite synaptic clefts	173	Takano et al. (2020)

APEX has also been used to determine cell-type-specific subcellular protein composition *ex vivo* (Dumrongprechachan et al., 2021) (Table 1). Utilizing a combination of AAV vectors and cell-type-specific conditional mouse lines, APEX2 was expressed in D1 or D2 types of medium spiny neurons of the striatum using Drd1-Cre and A2a-Cre mice, respectively (Dumrongprechachan et al., 2021). In addition, D1-type neurons were analyzed for their subcellular compartment-specific proteomic profiles using nucleus-APEX2 (H2B-APEX2), cytoplasm-APEX2 (APEX2-NES), and plasma membrane-APEX2 (Lck-APEX2). Consequently, this study successfully isolated cell-type-specific subcellular proteomic data from brain tissue samples, unveiling dynamic changes in the proteome of striatal neurons following chemogenetic activation of Gα_q_-coupled signaling cascades (Dumrongprechachan et al., 2021). Another research group employed a similar APEX-based approach to elucidate the protein compositions in midbrain dopaminergic neurons (Hobson et al., 2022) (Figure 1B; Table 1). APEX was expressed in dopaminergic (DA) neurons within the midbrain of DAT-IRES-Cre mouse using AAV vector. Subsequently, the cellular compartments such as the somatodendrites and axon terminal were isolated and analyzed by microdissection (Hobson et al., 2022). This approach determined the DA-specific presynaptic enriched proteins including DA neurotransmission and metabolism from the midbrain (Hobson et al., 2022). The application of APEX has further enabled the mapping of the cell-type-specific proteome and phosphoproteome of corticostriatal axons during neuronal development (Dumrongprechachan et al., 2022) (Table 1). APEX2 expression was induced in layer 5 cortical pyramidal neurons using *Rbp4*^Cre^ mice (Dumrongprechachan et al., 2022). Subsequently, the axonal proteome was isolated from the striatal lysates at various developmental stages (Dumrongprechachan et al., 2022). This study identified co-regulated proteins and phosphorylations in corticostriatal axons during neurodevelopment, which are linked to genetic risk for human brain disorders (Dumrongprechachan et al., 2022). Taken together, these findings demonstrated that iBioID and APEX approaches are highly effective for determining the cell-type-specific subcellular protein compositions *in vivo* and *ex vivo*.

## Mapping of cell-type-specific proteins at the cell surface, synaptic cleft and sites of cellular contact

Integrated neuronal systems communicate extensively through signaling in the synaptic cleft. This cell-surface signaling is pivotal in controlling nearly all aspects of neuronal development and physiology in the brain. Additionally, astrocytes and neurons form a unique cell adhesion structure called a “tripartite synapse,” suggesting that astrocytes’ direct involvement in synaptic formation and function (Allen and Eroglu, 2017; Takano and Soderling, 2021). However, comprehensive identification of molecular compositions at the cell-type specific interfaces *in vivo* has encountered significant limitations. This limitation stems from conventional biological approaches’ inability to capture protein components at synaptic clefts. Moreover, the characteristic attributes of cell-surface proteins, such as their low abundance, high hydrophobicity, and heterogeneity, further complicate this issue (Kuhlmann et al., 2018; Li et al., 2019).

Recent advancements in cell-surface proteomic profiling have enabled significant insights into neuronal synapse formation and function *in vitro* and *ex vivo* using HRP-based proximity labeling approaches (Loh et al., 2016; Cijsouw et al., 2018; Li et al., 2020; Shuster et al., 2022). For instance, HRP fused to known synaptic cleft proteins such as Lrrtm1, Lrrtm2, Slitrk3, or Neuroligin-2 (Nlgn2), have been employed for analysis of excitatory and inhibitory synaptic cleft proteomes in cultured neurons (Loh et al., 2016) (Figure 1B; Table 1). These studies have uncovered 199 glutamatergic and 42 GABAergic synaptic cleft proteins *in vitro.* Importantly, they found that a novel synaptic cleft protein Mdga2 plays a key role in inhibitory synapse formation by recruiting Nlgn2 to the postsynaptic site (Loh et al., 2016). Additionally, HRP fused to an excitatory cell adhesion molecule SynCAM 1 has deciphered several excitatory synaptic cleft proteins including receptor-type tyrosine phosphatase zeta (R-PTP-zeta) in cultured cortical neurons (Cijsouw et al., 2018) (Figure 1B; Table 1). In another study, the cell-type-specific expression of HRP fused to the N-terminal extracellular domain of CD2 (HRP-CD2) has been employed to discover novel cell surface molecules in *Drosophila* olfactory projection neurons *ex vivo* (Li et al., 2020) (Table 1). Recently, a new approach for cell-type-specific cell surface proteomics, termed *in situ* cell surface proteome extraction using extracellular labeling (iPEEL), has been developed (Shuster et al., 2022) (Figure 1B; Table 1). This approach extends the HRP-CD2-based proteome analysis by utilizing cell-type-specific conditional mouse lines. Using a transgenic mouse line (Pcp2-Cre), iPEEL identified 1,051 cell-surface proteins in Purkinje cells. Interestingly, it was revealed that mature cerebellar Purkinje cells and developing Purkinje cells exhibit substantial proteomic overlap. Additionally, different classes of cell-surface proteins exhibited selective enrichment at different developmental stages. Furthermore, they found that Armh4 plays an essential role in Purkinje cell dendrite morphogenesis by regulating endocytosis (Shuster et al., 2022).

A new approach known as TurboID-surface and Split-TurboID has emerged more recently for spatial proteomics on cell-type-specific cell surfaces and interfaces *in vivo* including astrocytic cell surfaces and tripartite synapses (Takano et al., 2020; Takano and Soderling, 2021) (Figure 1B; Table 1). TurboID-surface, fused to a glycosylphosphatidylinositol (GPI) anchor, has been delivered to cortical astrocytes using cell-type-specific AAV vectors (*GfaABC_1_D* promoter), facilitating the comprehensive profiling of astrocytic membrane proteins *in vivo*. Split-TurboID is composed of the N-terminal (N-TurboID) and C-terminal (C-TurboID) fragments of the TurboID surface. The cell-type-specific AAV vectors carrying N-TurboID and C-TurboID were expressed in mouse cortical neurons (human Synapsin I (*SynI*) promoter) and astrocytes (GFAP promoter), respectively. A functional TurboID is formed at the tripartite synaptic clefts once N-TurboID and C-TurboID fragments merge at the interface between neurons and astrocytes (Takano et al., 2020; Takano and Soderling, 2021). Super-resolution Stimulated Emission Depletion (STED) microscopy showed that astrocytic TurboID-surface and Split-TurboID were localized at the peri-synaptic sites *in vivo* (Takano et al., 2020). Using label-free quantitative LC–MS/MS analysis, TurboID-surface and Split-TurboID identified 178 and 173 extracellular proteins, respectively. One hundred and eighteen proteins were found to be common among these proteins, forming a tripartite proteome synapse with a high level of confidence (Takano et al., 2020). Notably, these spatial proteome approaches have unveiled a novel role for astrocytes in directly regulating the formation and function of inhibitory synapses through a new player NrCAM *in vivo*. Together, these studies provide novel insight into neuronal connectivity and brain function by mapping the protein composition in cell-type-specific synaptic cleft.

## Discussion

Deciphering the synaptic molecular landscape in specific cell types and synapses is crucial for understanding neural circuit formation and their roles in brain functions. Additionally, it’s essential for developing new therapeutic strategies based on the pathophysiology of neurological disorders characterized by synaptic dysfunction. However, given the considerable heterogeneity of neurons and glial cells in the brain, determining the molecular composition of specific cell types, along with their subcellular structures and synapses, continues to be a significant challenge in neuroscience.

Recent advancements in spatial proteomics, including FASS, APEX, and iBoID, have facilitated the analysis of protein components at targeted cellular and subcellular levels within the brain. These highly innovative approaches have uncovered numerous uncharacterized molecules in individual types of neurons, astrocytes, and specific synapses within brain tissue, each exhibiting unique molecular mechanisms. Employing these spatial proteome strategies will enhance our comprehension of various synaptic types, though they have a few restrictions to overcome. FASS approach shows a high level of specificity in selecting particular subcellular components, effectively reducing contamination (Biesemann et al., 2014; van Oostrum et al., 2023). This specificity is a consequence of synaptosome sorting properties, although it may result in lower throughput. Since neurons typically receive input from a wide variety of different presynaptic partners, the current FASS approach is restrained to targeting neurons at a cellular level, and may lead to an analysis that reflects only an averaged synaptic proteome. In contrast, APEX and iBioID allow spatial proteomic analysis at cell-type-specific levels combined with subcellular localization, including individual types of synapses from neurons and astrocytes in the brain, using cell-type-specific AAVs or transgenic mouse lines (Figure 1B). However, the *in vivo* application of both APEX and iBioID is constrained by certain limitations. The enzyme activity of APEX necessitates H_2_O_2_, which is cytotoxic and less amenable to labeling reactions in tissue. With iBioID, exogenous biotin administration for 7 days is necessary to achieve significant biotinylation labeling (Uezu et al., 2016; Spence et al., 2019). In addition, the enzyme activity of all BirA variants depends on ATP, which is typically present in low extracellular concentrations *in vivo* (Takano and Soderling, 2021). Despite these limitations, APEX and iBioID approaches have enabled successfully the identification of protein compositions in various neuron types—including glutamatergic neurons, dopaminergic neurons, and Purkinje cells as well as astrocytes (Table 1). The knowledge would provide deeper insight into how these different protein networks regulate individual neural circuits modulation and control various brain functions such as memory, learning, and emotion. Notably, iBioID accomplished subcellular protein profiling without causing cellular toxicity (Takano et al., 2020; Takano and Soderling, 2021). Furthermore, TurboID-surface and Split-TurboID have efficiently enabled the profiling of spatial protein networks in cell-type-specific synaptic clefts, showing particularly notable results in tripartite synapses (Takano et al., 2020; Takano and Soderling, 2021). Recent research has reported metabolic labeling methods as novel approaches for labeling nascent proteomes with cell type specificity in the mouse brain, such as MetRS and SORT (Alvarez-Castelao et al., 2017; Krogager et al., 2017; Tang and Chen, 2023). These methods utilize a methionyl-tRNA synthetase and an orthogonal pyrrolysyl-tRNA synthetase-tRNAXXX pair, thereby tagging newly synthesized proteins with non-canonical amino acids and click chemistry (Alvarez-Castelao et al., 2017; Krogager et al., 2017; Tang and Chen, 2023). The combination of these approaches with proximity labeling will offer more precise insights into the cell-type-specific synaptic proteome.

A recent study utilizing spatial transcriptomics has accomplished the single-cell tracing of developmental gene programs, leading to the differentiation of diverse cell types in the brain (Lein et al., 2017; Ratz et al., 2022). Similarly, these conceptual approaches are currently being adapted and optimized for proteomics. Integrating these applications with proximity labeling will advance spatial proteomic analysis, allowing for the tracking of the proteome within a single synapse in the future. These breakthroughs would enable the discovery of molecular mechanisms governing single neural circuit formation and function. Furthermore, recent evidence has increasingly indicated that the dysfunction of many synaptic genes significantly contributes to psychiatric and neurological disorders. Therefore, a comparative analysis of cell-type-specific synaptic proteomes is becoming increasingly essential in understanding the diverse pathogenesis of many neurological disorders. A large-scale interactome analysis of 14 risk genes including synaptic genes associated with ASD, using TurboID, has revealed a wealth of common cellular functions and molecular interactions related to the pathomechanisms of ASD (Gao et al., 2023). This dataset is expected to contribute to the establishment of new diagnostic and therapeutic methods targeting common molecules in ASD pathology. Taken together, advancing these studies further could eventually involve investigations exploring variations in cell-type-specific synaptic proteomes among different states of neuronal cells and glial cells *in vivo*. The consolidation of this accumulated data into a database is expected to substantially progress our comprehension of the brain’s information processing mechanisms and pharmaceutical development.

## Author contributions

YI: Conceptualization, Visualization, Writing – original draft, Writing – review & editing. SN: Writing – review & editing. TT: Conceptualization, Funding acquisition, Visualization, Writing – original draft, Writing – review & editing.
